# Metal Ion-Loaded Nanofibre Matrices for Calcification Inhibition in Polyurethane Implants

**DOI:** 10.3390/jfb8030022

**Published:** 2017-06-23

**Authors:** Charanpreet Singh, Xungai Wang

**Affiliations:** 1 Australian Future Fibres Research and Innovation Centre, Institute for Frontier Materials, Deakin University, Geelong, VIC 3216, Australia; c.singh@deakin.edu.au; 2 School of Textile Science and Engineering, Wuhan Textile University, Wuhan 430073, China

**Keywords:** calcification, magnesium, metal ion, Von Kossa method, Alizarin red S staining, anti-calcification, nanofibre matrix, hydroxyapatite

## Abstract

Pathologic calcification leads to structural deterioration of implant materials via stiffening, stress cracking, and other structural disintegration mechanisms, and the effect can be critical for implants intended for long-term or permanent implantation. This study demonstrates the potential of using specific metal ions (MI)s for inhibiting pathological calcification in polyurethane (PU) implants. The hypothesis of using MIs as anti-calcification agents was based on the natural calcium-antagonist role of Mg^2+^ ions in human body, and the anti-calcification effect of Fe^3+^ ions in bio-prosthetic heart valves has previously been confirmed. In vitro calcification results indicated that a protective covering mesh of MI-doped PU can prevent calcification by preventing hydroxyapatite crystal growth. However, microstructure and mechanical characterisation revealed oxidative degradation effects from Fe^3+^ ions on the mechanical properties of the PU matrix. Therefore, from both a mechanical and anti-calcification effects point of view, Mg^2+^ ions are more promising candidates than Fe^3+^ ions. The in vitro MI release experiments demonstrated that PU microphase separation and the structural design of PU-MI matrices were important determinants of release kinetics. Increased phase separation in doped PU assisted in consistent long-term release of dissolved MIs from both hard and soft segments of the PU. The use of a composite-sandwich mesh design prevented an initial burst release which improved the late (>20 days) release rate of MIs from the matrix.

## 1. Introduction

Calcification is a normal, or rather physiological event (physiological mineralization) in the formation of hard tissues (bones and teeth). The main product of calcification is a hydroxyapatite (HAP) crystal, Ca_10_(OH)_2_(PO_4_)_6_ which provides structural integrity to the bone. Under normal conditions, calcium exists in a metastable equilibrium with dissolved phosphate species [1]. Any skewness in equilibrium can result in calcification of functional soft tissues and synthetic implant materials in a process known as pathologic calcification [2,3,4]. Pathologic calcification can be of two types; dystrophic and metastatic [5]. Dystrophic calcification is commonly observed as calcific deposits in cells and the extracellular matrix of damaged or diseased tissues [6]. In synthetic implants, dystrophic calcification is dependent on numerous factors, namely material properties, device design, surface chemistry, host biological system, and stress concentration etc. On the other hand, in metastatic calcification, the calcific deposits start forming deep within the biological tissue (intrinsic calcification). This occurs due to disruption in mineral metabolism, or disproportionate calcium or phosphorus levels [7].

Deposition of calcium crystals has been widely reported in soft tissue implant devices like heart valves, vascular grafts, stents, cardiac assist devices, and blood pumps etc. [7,8,9,10]. Calcification can lead to structural deterioration of implant materials via stiffening, stress cracking and other structural disintegration mechanisms, and the effect can be critical for implants intended for long-term or permanent implantation. Polyurethanes (PU)s are extensively used synthetic materials for heart valve implants and artificial blood pump applications, and hence calcification is a major concern for their intended application. Calcification reduces the fatigue life and elasticity of the PU, which ultimately can translate into poor long-term performance of the implant [5]. Hard HAP crystals can induce fracture failures, even in metallic stent implants [11]. The fatigue is much more severe in the case of angulated small diameter lesions where calcified hard regions cyclically rub against already stressed stent struts [11]. The hardness of calcium deposits has been found to be similar to that of metals such nickel and iron (710 MPa or 72.4 Vickers) [12], which directly indicates the severity of metal fatigue under continuous pulsatile conditions.

Despite the clinical importance of the problem, the mechanism of calcification is not yet completely understood [13,14,15]. Furthermore, there is no effective treatment therapy still available. Calcification is a multifactorial process, and hence an effective therapeutic strategy may require drug action at different stages of calcium phosphate deposition [13,16]. A therapeutic approach involves implantation of a controlled release drug delivery system that facilitates local, site-specific anti-calcification. The therapeutic applications of metal ions (MI)s in regenerative medicine and tissue engineering fields are readily increasing. This increased interest is owed to its low cost, increased stability, and, relatively lower risk than proteins or genetic engineering techniques [17]. Therefore, numerous strategies of MI release from polymeric matrices or scaffolds are being designed. PU qualifies well for versatile medical applications [18,19,20], owing to its microstructure, which allows easy incorporation of functional groups and bulk modifications [21]. Several investigations have been performed using MI release matrices showcasing controlled release and improved therapeutic action at the treatment site [22,23,24]. However, the design of MI-loaded polymeric matrices is a critical task, as high MI concentration can lead to significant systemic toxicity [17]. The design features include an optimum degree of MI loading and their controlled release at the intended treatment site.

In blood serum, the Mg/Ca ratio of about 0.3 is naturally sufficient to prevent HA precipitation in the body fluids. A similar role is performed by Mg ions in sea water, by controlling calcite (CaCO_3_) growth rate [25]. Aluminium (Al) ions can also delay HA formation by adsorbing onto the surface of growing HA crystals. Al ions slow the direct precipitation of HA, the transformation of amorphous calcium phosphate (ACP) to HA, and the growth of HA seed crystals, all in a dose-related manner. While the divalent Mg ion (Mg^2+^) is believed to enter the forming HA embryonic nuclei, the smaller, trivalent Al ion (Al^3+^) is believed to bind and block active growth sites on the surface of forming HA crystals, thereby slowing apatite proliferation. Also, aluminium chloride (AlCl_3_) has been claimed to prevent elastin fibre calcification through permanent structural alteration in the elastin molecule [7]. Ferric ions (Fe^3+^) have also been reported to inhibit the progression of calcification via the generation of reactive oxygen species and oxidative stress mechanisms in vascular tissues [26]. Vasudev et al. proposed that Fe^3+^ ions slowed down the calcification process by the inhibition of HAP formation, while Mg^2+^ ions disrupted the growth of HAP crystals by replacing Ca^2+^ ions [27]. It was also hypothesised that both Fe^3+^ and Mg^2+^ ions can inhibit formation of alkaline phosphate which acts as a substrate for HAP crystal binding.

Magnesium is the second most abundant intracellular cation (next to potassium), and the fourth most abundant cation in the body [28,29]. Magnesium is involved in many essential physiological, biochemical and cellular processes regulating cardiovascular function, such as contraction and dilation, growth and inflammation, production of vasoactive agents, and protein and nucleic acid synthesis [30,31]. Magnesium is considered nature’s physiological calcium blocker [32]. Direct relationships between low serum levels of magnesium and vascular calcification has been reported widely in both human and experimental animal studies [33,34,35,36,37,38,39]. Several in vitro studies have shown that magnesium’s inhibitory effect on HAP formation and precipitation, as well as on the calcification process [40,41,42], while magnesium deficiency appears to promote vascular calcification [43]. Magnesium ions can stabilize ACP and thus inhibit the formation of calcium-acidic phospholipid–phosphate complexes in metastable calcium phosphate solutions [44,45]. Bennett et al. found that magnesium was also able to inhibit calcium pyrophosphate dihydrate crystal formation in-vitro [46]. In another study, aortic segments of rats were incubated in the absence and presence of MgCl_2_ in a calcification medium with elevated phosphate concentrations [47]. The degree of calcification was significantly decreased in the aortic rings incubated in the presence of magnesium. The exact underlying antagonist mechanism of magnesium has not been resolved. However, the reported studies support the use of Mg^2+^ ion as a potential drug delivery agent for reducing implant calcification in a more physiologic manner. Magnesium-based compounds also have the additional advantage of being much cheaper to use than some newer alternative drugs. However, the optimal level of release rate and the Mg^2+^ ion concentration are some concerns that remain to be determined.

In this study, we reported on the design and comprehensive performance evaluation of MI-loaded nanofibre matrices for the inhibition of calcification, in an in vitro study. MIs selected for the current study (Mg^2+^ and Fe^3+^) were based on their easy availability in the form of commercial salts (MgSO_4_, MgCl_2_, FeCl_3_), minimal toxicity levels, and fair solubility in organic solvents used for PU nanofibre spinning.

## 2. Results and Discussion

### 2.1. Morphological Analysis 

**Figure 1:**
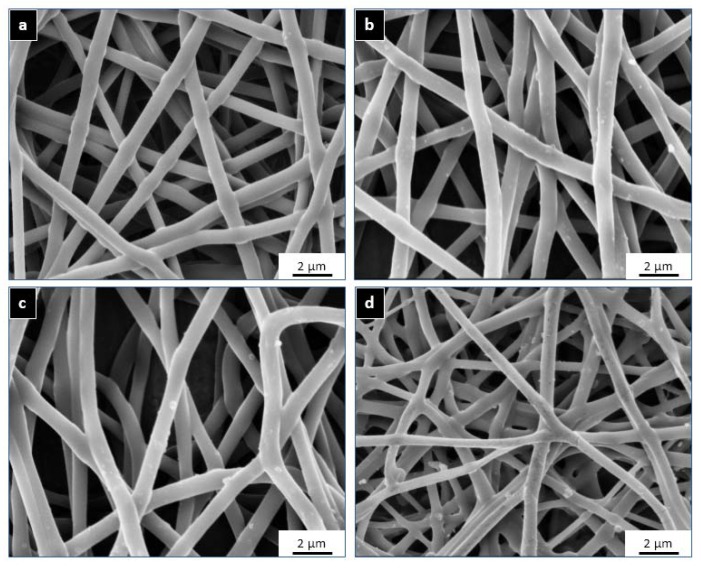
Scanning electron microscopy (SEM) micrographs of electrospun (**a**) Polyurethane (PU); (**b**) MgSO_4_-loaded sample (PU-MS); (**c**) MgCl_2_-loaded sample (PU-MC); and (**d**) FeCl_3_-loaded sample (PU-FC) films.

The surface analysis of all the matrices (PU: undoped control sample, PU-MS: MgSO_4_-loaded sample, PU-MC: MgCl_2_-loaded sample, PU-FC: FeCl_3_-loaded sample) showed a random fibre distribution on the surface with average fibre diameter of 820 ± 110 nm (Figure 1). There was insignificant effect of magnesium salt (MgCl_2_ and MgSO_4_) doping on fibre configuration (800 ± 105 nm and 795 ± 100 nm, respectively) while FeCl_3_ incorporation resulted in relatively finer fibres (750 ± 180 nm). The presence of very few salt particles on the PU-MI film surface indicated nearly homogenous dissolution of metal salts in the polymer-solvent system.

### 2.2. Fourier Transform Infra RedAnalysis

**Figure 2:**
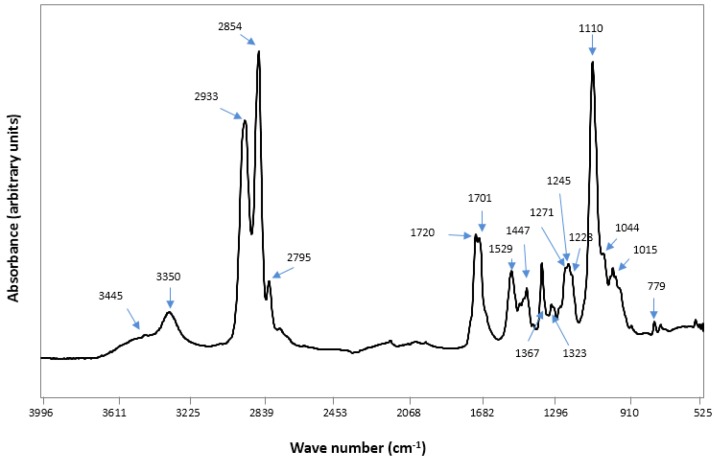
Fourier transform infra red (FTIR) spectrum of electrospun polyurethane film.

The characteristic Fourier transform infra red (FTIR) spectrum of the control PU sample depicted hard segments (HS) or urethane (779, 1044, 1228, 1529, 1701, 1720, 3323, 3445 cm^−1^), and soft segments (SS) or ether (1110, 1367, 2795, 2854, 2933 cm^−1^) absorption bands (Figure 2). The donor groups (free N-H of urethane) and acceptor groups (free C=O of urethane, free C-O-C of ether) in the spectrum indicated several types of H-bonding which could occur at these sites between HS and SS, and also within HS [48].

**Figure 3:**
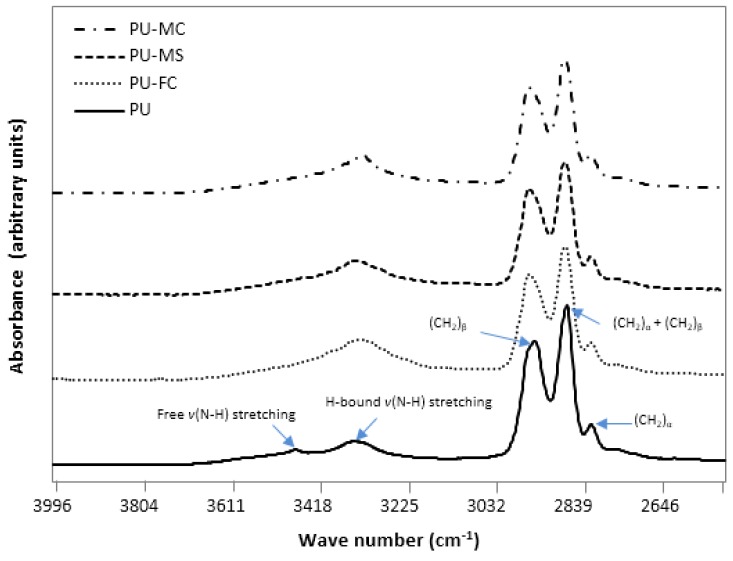
FTIR spectrum of electrospun control PU and metal ion (MI) loaded PU films in the 2500–4000 cm^−1^ frequency range.

**Figure 4:**
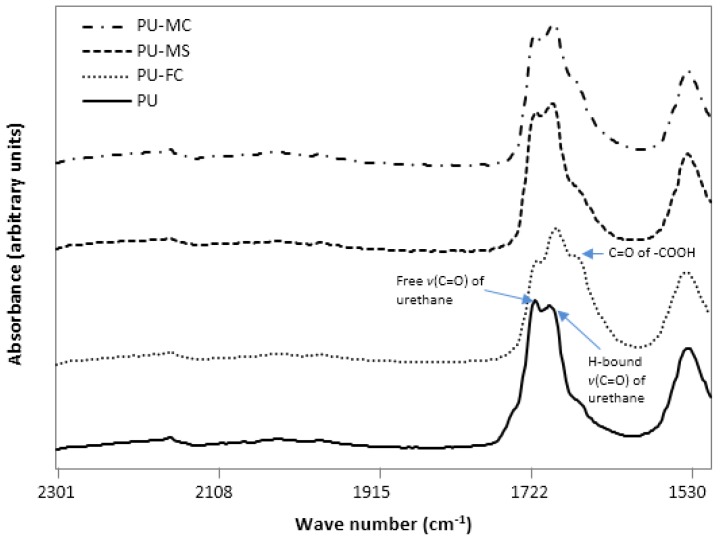
FTIR spectrum of electrospun control and MI-loaded PU films in the 1500–2300 cm^−1^ frequency range.

**Figure 5:**
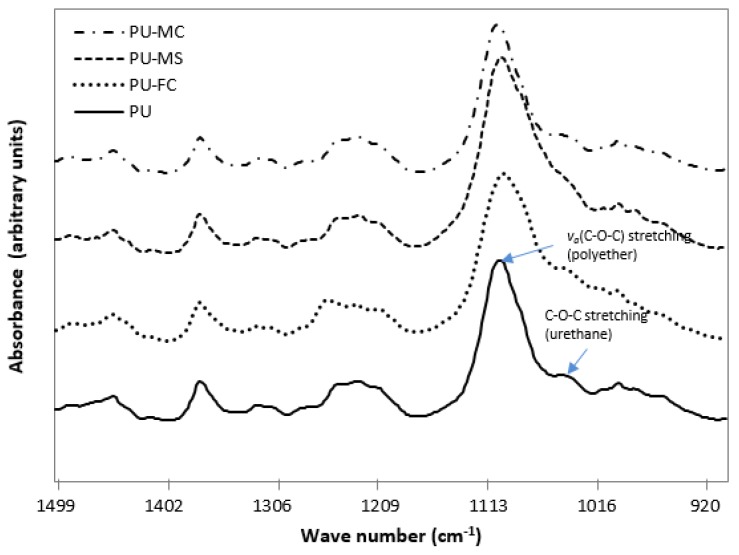
FTIR spectrum of electrospun control and MI-loaded PU films in the 900–1500 cm^−1^ frequency range.

The overall changes which occurred in HS after metal salt doping indicated that there was a simultaneous decrease (free C=O and free N-H) and increase (H-bonded C=O and H-bonded N-H) in its constituent units (Figure 3, Figure 4 and Figure 5). This suggested that an increased phase separation (or increased inter-urethane H-bonding) was favoured after MI incorporation in PU. The observed changes in the intensity of both ether band (1110 cm^−1^) and specific urethane bands (3350, 1701 cm^−1^) were, respectively, indicative of MI interaction with both SS and HS of the PU. Generally, the PU chain scission induced by specific metals was associated with the generation of new organic compound bands viz. carboxylic acid (1700–1630 cm^−1^; 930 cm^−1^), aldehydes (1740 cm^−1^), alcohols (3650 cm^−1^, 1420–1260 cm^−1^), and crosslinking reactions (1175 cm^−1^) [49,50]. However, in our experiments, such degradation products were not observed after magnesium salt doping, indicating no significant structural degradation of PU. However, doping of ferric salt (FeCl_3_) may have resulted in HS chain scission, as indicated by the formation of a carboxylic acid band at 1670 cm^−1^ (Figure 4). MI doping in PU resulted in increased phase separation between HS and SS, as observed from increased inter-urethane H-bonding within hard domains of the PU matrix. FTIR analyses revealed that MIs were incorporated in both the HS and SS of the PU, but the exact state (amorphous/crystalline) of these ions was not known.

### 2.3. Mechanical Properties

**Figure 6:**
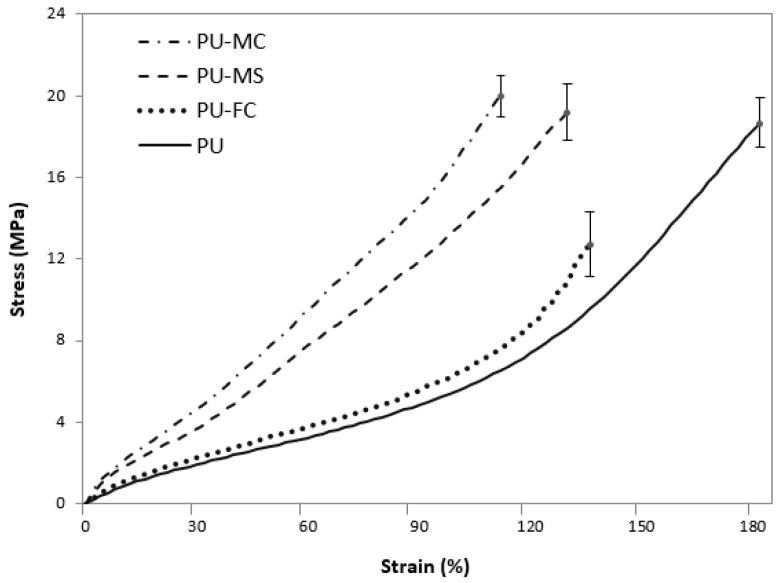
Stress-strain relation of control and MI loaded PU films.

The mechanical characteristics of PU films are primarily controlled by their chemical compositions, i.e., the ratio between SS and HS, and physical cross-links formed by H-bonding (urethane-urethane or urethane-ether). As depicted by changes in urethane H-bonding (Section 2.2), similar changes are expected in the mechanical properties of PU after MI doping. Figure 6 shows the tensile behaviour of undoped and MI-doped PU films. There was a clear difference in the effect of MI type on the tensile behaviour. PU-FC exhibited a significant decrease (31%) in peak strength compared to undoped PU, this was attributed to the observed chain scission phenomenon caused by Fe^3+^ ions (Section 2.2). Both PU-MC and PU-MS films exhibited minimal changes in strength. However, the higher stiffness of Mg^2+^-doped PU than undoped-PU was observed, and attributed to higher crosslinking within the polymer chains [51,52].

### 2.4. Metal Ion Release

For thin film membranes, drug release versus time curves are often analysed using the power law [18,53] as in Equation (1) below:

*M_t_*/*M*_∞_ = *k* × *t^n^* (1)

where *k* is a constant related to the characteristics of the matrix system, and is a measure of the release rate; *n* is a diffusional exponent which is characteristic of the mode of drug transport through the matrix (*n* = 0.5 indicates Fickian diffusion and *n*=1 indicates a zero-order release; 0.5 < *n* < 1 indicates non-Fickian diffusion; *t* is time) [54,55,56].

**Figure 7:**
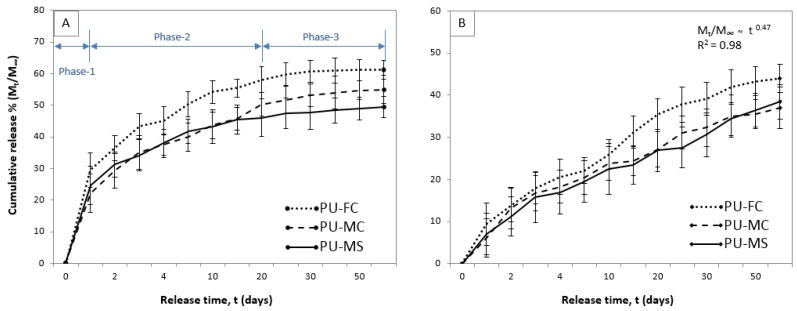
Release profile of MIs from PU-MI matrices in (**A**) solid-sandwich; and (**B**) composite-sandwich configurations.

The release profiles of MIs from solid-sandwich films exhibited a three phase release behaviour (Figure 7A). The initial burst release (0–24 hr or Phase 1) signified instant dissolution of surface deposited salt crystals. The dissolution of even a small portion of these crystals can cause burst release by creating new vacant pore sites for the elution media to come in contact with sub-surface particles [57]. Therefore, the degree of burst release was different for all three salts, with FeCl_3_ exhibiting higher burst percentages (31%) than magnesium salts (MgCl_2_ = 22% and MgSO_4_ = 24%). This was attributed to the highly hygroscopic nature of FeCl_3_ which instantly attracts water molecules to interact with the film surface and hence dissolve the sub-surface salt particles. After the surface dissolution of drug is exhausted, Phase 2 of drug release initiates, which is primarily governed by diffusion of the drug from polymeric matrix. As evident from the release profile, the release rate for second phase (Day 2–20) was slow and consistent (40–80 μg/cm^2^/day). This phase represented MI transport through soft domains (or SS) of the polymer matrix, which is dependent on the degree of PU phase separation. A nearly consistent release of MIs over a long time period (18 days) in the second phase indicated a sufficiently high content of salt particles present in both soft and hard domains of the polymer. The MI release in this phase was considered to be based on the physiochemical nature of both HS and SS, with the former acting as a micro-reservoir while the latter acted as ion transport channels [58,59,60]. Based on Equation (1), a power law fit was performed and the diffusion exponent *n* was calculated for second phase release profiles of all MIs. In order to explain the drug release mechanism by diffusion only, the value of *n* should be nearly equal to 0.5, but in our experiments the value of *n* < 0.5 was observed, indicating that the diffusion mechanism of MIs release suffered some retardation in the system. These observations matched the *n* values observed in the release of copper and silver ions from MI-silicone composites [22], indicating that the power law provided a limited insight to the different mechanisms involved in the MI release kinetics from non-degradable monolithic matrix systems. By the end of second phase (20 days), nearly half (45–55%) of MIs were released. This was followed a very slow (6–8 μg/cm²/day) but still consistent Phase 3 over rest of the experimental duration (day 20–60), suggesting a long-term ion release from monolithic polymer matrices, as observed elsewhere [22]. PU-FC exhibited insignificant release during Phase-3 (<2 μg/cm^2^/day) which was virtually undetectable. Overall, the MI release profiles of MI-PU films appeared to be connected with PU microdomain structures, with HS acting as micro-reservoirs which helped to maintain long-term ion release. The continuous release of MIs also indicated that there was sufficiently high availability of free MIs which can undergo diffusion through the PU microstructure. This may be attributed to limited number of available sites (1–2 ions per chain) in the SS domain for MI complex formation [51].

Figure 7B shows the MI release of composite electrospun films exhibiting significantly reduced burst release profile compared to composite films. This improvement can be attributed to the obvious low surface area available for MI dissolution in a composite mesh compared to a normal MI loaded mesh. Also, as the un-doped PU nanofibres were hydrophobic, they could markedly inhibit the initial invasion of the release medium into the MI loaded nanofibre mesh. The contact area of the release medium thus would be restricted in a superficial region of the nanofibre mesh. This made the un-doped PU nanofibres behave similarly to an external “barrier mesh” without even completely covering the MI-doped fibres. Interestingly, this partial inhibition of burst release resulted in a nearly consistent (30–50 μg/cm^2^/day) long-term (day 20–60) release of MIs from composite films matching the Fickian diffusion (*n* = 0.44–0.48, *R*^2^ = 0.98) principle.

### 2.5. Calcification

**Figure 8:**
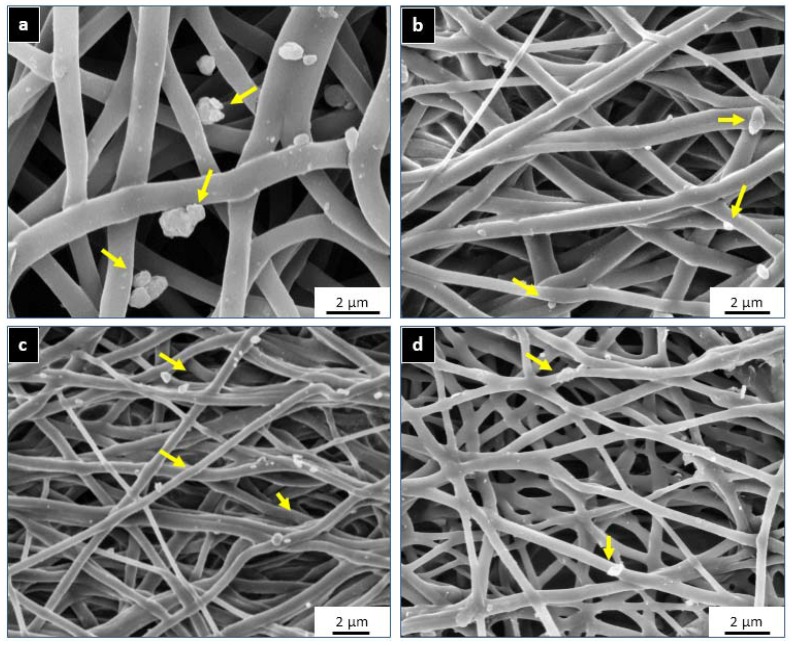
SEM micrographs of (**a**) PU; (**b**) PU-MS; (**c**) PU-MC; and (**d**) PU-FC films after calcium solution incubation for 60 days. Calcium deposits are visible as randomly segregated crystals adhering to fibres.

**Figure 9:**
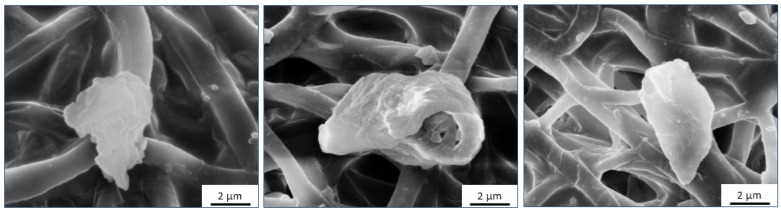
SEM micrographs of large calcium deposits on the surface of Control PU film after calcium solution incubation for 60 days.

All the MI doped PU matrices (PU-FC, PU-MC, PU-MS) significantly inhibited calcific deposition compared to the control PU matrix. Scanning electron microscopy (SEM) imaging indicated that the calcific deposits were randomly distributed on the film surface but with varied number and size. The deposits on PU films were observed as firmly bound spherical crystals (average diameter = 2–6 μm, Figure 8 and Figure 9) similar to those observed by several investigators previously [61,62,63,64,65]. These deposits were not affected by subsequent rinsing and remained adherent in localised fibre interstices as expected in the calcification of porous mesh structures [64,66]. In case of MI-PU films, similar deposits were observed, but in very sparsely populated and submicron size (average diameter = 0.2–0.8 μm) configurations. The small size of calcific deposits represented the inhibition of calcium-phosphate crystal formation in the initial stages of nucleation itself [67]. Thus, it appears evident that the released Mg^2+^ and Fe^3+^ ions prevented calcification by interrupting the formation of proper HAP crystals on the film surface, as proposed by Vasudev et al. [27].

**Figure 10:**
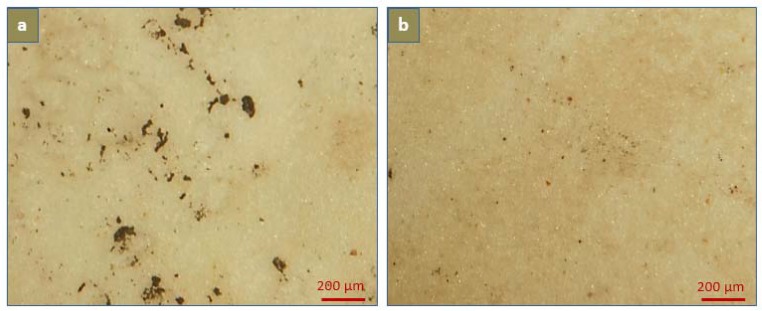

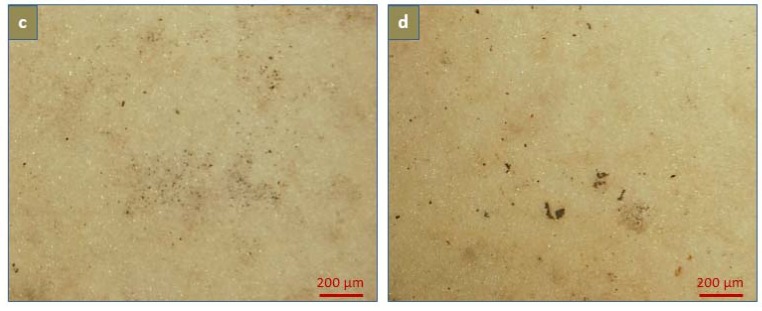
Light microscopy of (**a**) PU; (**b**) PU-MS; (**c**) PU-MC; and (**d**) PU-FC films after Von Kossa staining and calcium solution incubation for 60 days. Dark black-brown spots indicate aggregated calcium deposits.

**Figure 11:**
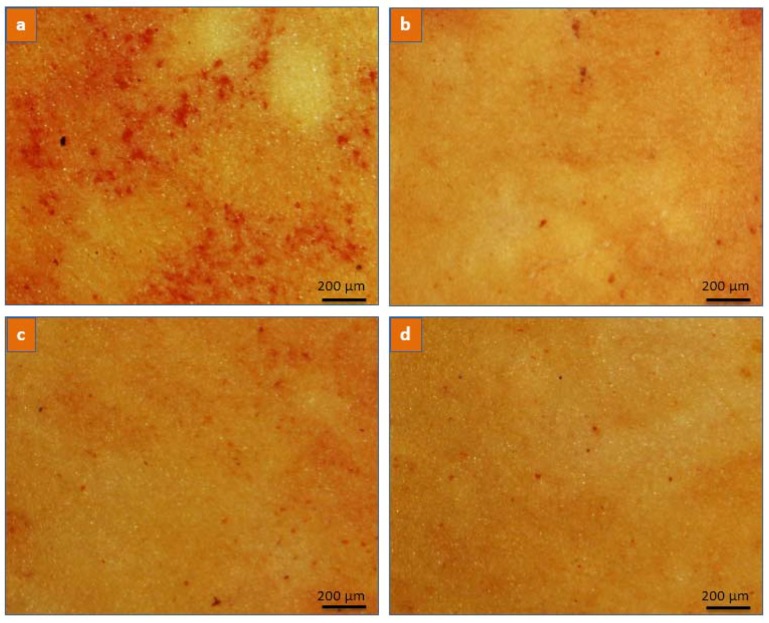
Light microscopy of (**a**) PU; (**b**) PU-MS; (**c**) PU-MC; and (**d**) PU-FC films after Alizarin Red staining and calcium solution incubation for 60 days. Dark red-orange spots on the surface indicate aggregated calcium deposits.

Von Kossa and Alizarin Red S staining further confirmed the antagonist effect of Mg^2+^ and Fe^3+^ ions on calcium crystal formation (Figure 10 and Figure 11). Microscopic examinations of the stained undoped PU films showed areas of calcification distributed randomly on the surface with average spot size of 0.3 mm × 0.3 mm. The large calcification areas were at a later stage after initiation of calcium crystal formation, which involves simultaneous cascaded deposition of both Ca^2+^ and PO_4_^3−^ ions. On the contrary, no observable calcium aggregated regions were observed in MI-doped PU films, indicating the absence of fully developed HAP crystals or the Ca^2+^ ion cascading phenomenon.

**Figure 12:**
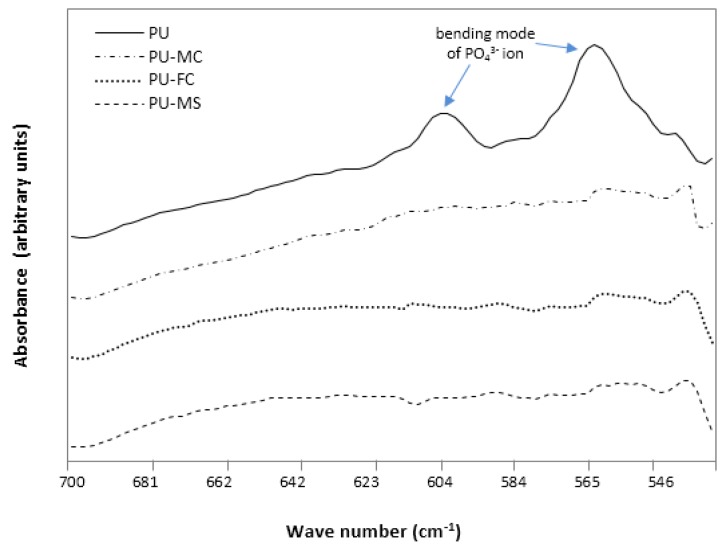
FTIR spectrum of control PU and MI loaded PU films after 90 days of incubation in calcification solution.

The undoped PU matrices showed significant calcified regions on the surface, even in the absence of dynamic stress, owing to the highly porous structure of nanofibre films. FTIR analysis revealed the absence of any intrinsic calcification in the presence of MIs, while undoped-PU exhibited traces of calcification at the microstructure level. Since calcification involves the nucleation of both calcium and phosphate ions [66], the level of calcification was detected corresponding to the phosphate ion (PO_4_^3−^) as the O-P-O bond bending mode at 603 and 562 cm^−1^ bands [68,69,70]. The FTIR spectrum showed prominent peaks at 603 and 562 cm^−1^ in PU films, while peaks were absent in MI-PU films (Figure 12). The reason behind absence of these peaks on all PU-MI films could be attributed to the assumption that calcification was limited to the surface only and did not interact with the microstructure of the polymer.

While comparing the magnesium ion (Mg^2+^) with ferric ion (Fe^3+^), it appears that the divalent magnesium had less ‘hard character’ than trivalent ferric ion. Their ionic radii (Fe = 68–71 picometer, Mg = 72 picometer) are similar. However, an extremely important characteristic of MIs to be determined for their use in the PU matrix was their oxidation tendency (or redox potential) [71,72,73]. The redox potential of Fe^3+^ ions (0.77 V) is very high compared to Mg^2+^ ions (−2.36 V). This indicates a higher tendency of Fe^3+^ ions to degrade the PU structure via the MI oxidation mechanism (Section 2.3), as metal ions with a redox potential higher than 0.7 have been found to potentially degrade PU elongation and tensile properties [71,74]. Therefore, the use of magnesium ions for inhibiting PU calcification appears to be a viable option.

## 3. Materials and Methods 

Medical grade segmented thermoplastic polyurethane (PU), Tecoflex^®^ was purchased from Lubrizol Advanced Materials, Inc. (Ohio, OH, USA) The solvents N,N-dimethylformamide (DMF) and Tetrahydrofuran (THF), 1,1,1,3,3,3-hexafluoro-2-propanol (HFIP), and metal salts (magnesium sulphate, magnesium chloride, and ferric chloride) were obtained from Sigma-Aldrich Co. (Castle Hill, Australia). All other chemicals were of analytical grade purchased from Sigma-Aldrich Co.

### 3.1. Electrospinning and MI Loading

**Figure 13:**
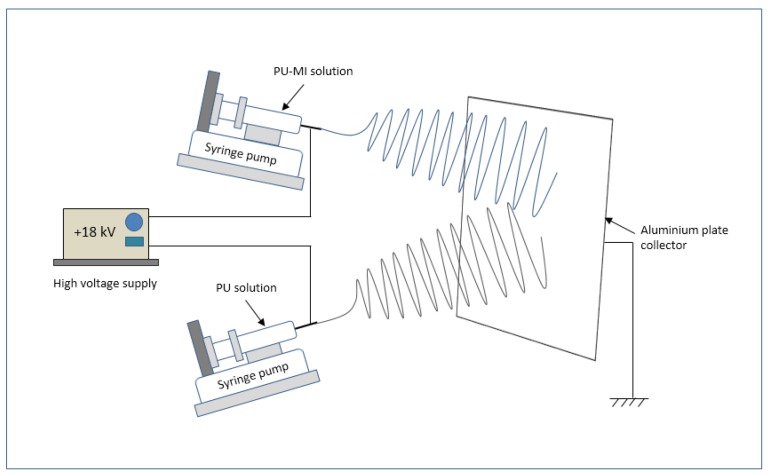
Schematic of electrospinning setup for developing metal salt-loaded PU films.

**Figure 14:**
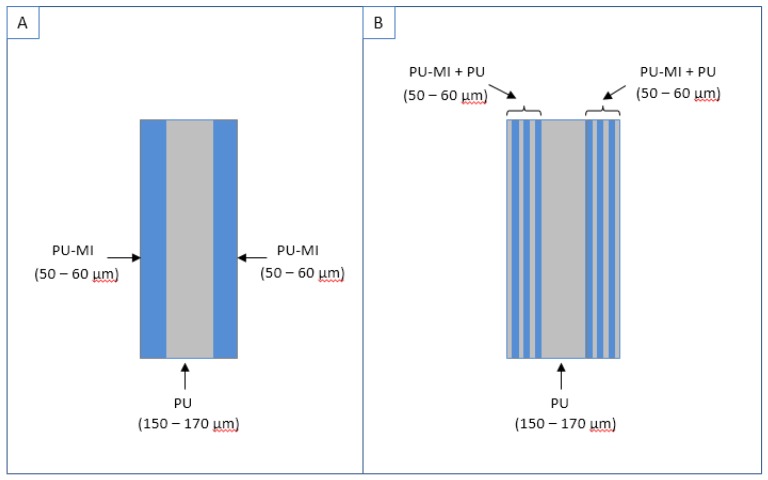
Representation showing cross-section of (**A**) solid-sandwich; and (**B**) composite-sandwich electrospun films.

PU pellets were dissolved in DMF/THF (40/60, v/v) solution at concentration of 11% (w/v). Polyurethane-metal ion (PU-MI) solutions were separately prepared by blending metal salts in 11% (w/v) PU solution in specific ratios to obtain an equal concentration of MIs in each PU-MI solution (Table 1). Electrospinning was done using a syringe pump (Harvard Apparatus, MA, USA) and a high voltage power supply (Spellman High voltage, Hauppauge, NY, USA) at +18 kV potential (Figure 13). Based on experimental characterization requirements, two geometrical configurations of electrospun samples were prepared (Figure 14): (a) thick films (100–150 μm) of undoped-PU and PU-MI for FTIR, morphological, and mechanical characterization; (b) solid-sandwich and composite-sandwich films (100–150 μm thickness) for MI release and calcification studies. Composite-sandwich films were produced by loading PU and PU-MI solutions in separate syringes and simultaneously electrospinning on both sides of PU film. Solid-sandwich films were produced by spinning only PU-MI solutions on both sides of the PU film.

### 3.2. Surface Morphology Analysis 

Specimens (5 mm × 5 mm) were cut from PU and PU-MI films. The samples were gold-coated (Baltec SCD50 sputter coater, Leica Microsystems Pty Ltd., Macquarie Park, Australia) and then viewed at different magnifications under a scanning electron microscope, SEM Neoscope (JCM-5000, JEOL Pty Ltd., Frenchs Forest, Australia) at a 15-kV accelerating voltage. Fifteen images per sample type were analysed and the average diameter of the fibres was calculated from 20 random measurements per image. The diameter was measured manually using Image J software (NIH, MD, USA, 2008). The results were expressed as mean±standard deviation (Table 1).

### 3.3. Mechanical Characterisation 

The tensile properties of films were determined using a tensile tester (Model 5967, Instron Pty Ltd., Bayswater, Australia). The films were cut into rectangular strips (10 mm × 5 mm, *n* = 20) and tested at speed of 20 mm/min up to breaking strength, using a load 100 N load cell. Each strip was preconditioned by application of 25 cycles at 50 mm/min and 10% strain limit. The ultimate tensile strength, breaking elongation and Young’s modulus were obtained from the stress-strain curves.

### 3.4. MI Release

The MI release measurements were performed in PBS buffer (pH 7.4) medium. MI-PU film samples (1 cm × 1 cm, *n* = 10) were immersed in small glass bottles filled containing 5 mL of the medium and swirling at 100 rpm on a rotating shaker. At appropriate intervals, 2 mL of medium was withdrawn from the bottle and tested for released MI content over a period of 60 days. An equal volume of the fresh dissolution medium was added to the bottle again in order to maintain a constant volume. The amount of MIs (Fe^3+^ and Mg^2+^) released from MI-PU films was measured on an atomic absorption spectrometer (AAS, SpectrAA 140, Varian Inc., Palo Alto, CA, USA) equipped with a flame furnace. A hollow cathode lamp was used for the measurement of Fe^3+^ at 248.3 nm and Mg^2+^ at 202.6 nm wavelengths. An air/acetylene mixture was used as the flame gas with a flow rate of 3.5 L/min for air and 1.5 L/min for acetylene. Initially, a linear calibration curve for both MIs was obtained in the range 2–10 mg/L (R^2^ > 0.99) followed by sample testing. The released MI content was expressed as cumulative release in μg/cm^2^. Each PU-MI sample release was studied over seven parallels and the final reading was calculated average of all parallels.

### 3.5. In-Vitro Calcification 

The calcification solution was prepared using the following formulation: [Na^+^] = 136.8 mM, [Cl^−^] = 144.5 mM, [Ca^2+^] = 3.87 mM, [HPO_4_^2−^] = 2.32 mM, [K^+^] = 1.16 mM. The solution was buffered in 50 mM Tris buffer at pH = 7.4 and maintained at room temperature. This preparation recipe gave a calcium concentration similar to the mean level of physiological calcium in serum, with a ratio of Ca/PO_4_ = 1.67, identical to hydroxyapatite (HAP). The calcification tests were conducted by incubating PU and MI-PU films in the calcification medium. Four specimens (20 mm × 10 mm) were cut from each type of electrospun film type. Each of these specimens were put into four separate bottles, each containing 20 mL of the calcification solution and incubated for period of 60 days. The bottles were sealed and placed in a water bath shaker (100 rpm) at 37 °C. All specimens were inspected daily during the entire period of incubation. The calcification solutions were replaced every five days to maintain electrolyte concentrations and to keep the pH constant. After 60 days, the film samples were taken out and rinsed four times in distilled water to remove any residual solution and loosely attached deposits. The samples were then dried in a vacuum oven for 48 hr at room temperature.

#### 3.5.1. Degree of Calcification

Calcified samples were observed for degree of calcification by using Von Kossa and Alizarin Red S staining techniques. For Von Kossa staining, the films were stained by incubating in 5% silver nitrate solution in glass beaker for 45 min [75]. During this time, the solution was exposed to bright light using a 60 watt lamp while the beaker was covered with aluminium foil, to reflect maximum light. This was followed by rinsing of specimen films in distilled water three times. The samples were then immersed in 5% sodium thiosulfate for 5 min followed by another again. Finally, the samples were dehydrated and mounted on coverslips for microscopic analysis. The deposition of HAP crystals was visualised as black-brown spots on the film surfaces. Separately, the samples were also analysed with Alizarin Red S method. Alizarin Red S dye (2 gm was dissolved in deionised water (100 mL). The pH was adjusted to 4.1–4.3 with 10% ammonium hydroxide. Since pH is a critical factor, the solution was made fresh every time and checked for pH regularly. Specimen films were stained in the solution for 4–5 min and the reaction was observed microscopically until the orange colour started appearing. The samples were taken out and blotted carefully to remove excess dye on the surface. Calcium deposits appeared as a dark red-orange colour.

### 3.6. Infrared Spectroscopy Analysis

Infrared spectra of films were recorded with an attenuated total reflectance (ATR) FTIR spectrophotometer (VERTEX 70, Bruker, Billerica, MA, USA). Each spectrum was obtained in absorbance mode at 32 scans per piece between 4000 and 500 cm^−1^ at intervals of 1 cm^−1^ and with a resolution of 4 cm^−1^.

## 4. Conclusions

This study demonstrated the potential of controlled therapeutic delivery of Mg^2+^ ions in a PU nanofibre matrix for inhibition of calcification. Since, replenishment of the drug into the matrix after exhaustion is not possible in monolithic systems, the long-term (1–2 years) performance of these functional PU matrices is warranted. The role which Mg plays in the prevention of calcium crystal deposition on matrix surface can significantly lower the risk of embolic stroke events caused by a dislodged HAP crystal specifically in vascular implants, as well enhance long-term implant functionality.

## Figures and Tables

**Table 1:** Sample type description for each metal salt loaded electrospun PU covering.

Sample Name	GSM (gm/m^2^)	Thickness (μm)	Fibre Diameter (nm)	PU % (w/v)	Metal Salt	Metal Ion Content in Salt (w/w)	Metal Ion Content in PU (w/w)
Control PU	130 ± 12	150 ± 10	820 ± 90	11	-	-	-
PU-MC	125 ± 11	140 ± 12	800 ± 105		MgCl_2_	0.26	0.108
PU-MS	134 ± 10	135 ± 12	795 ± 100		MgSO_4_	0.2	0.109
PU-FC	124 ± 15	145 ± 10	750 ± 180		FeCl_3_	0.34	0.107
